# Supplementary material for: Rapid Responses of Greenhouse Gas Emissions and Microbial Communities to Carbon and Nitrogen Addition in Sediments
Source: Microorganisms. 2024 Sep 25;12(10):1940. doi: 10.3390/microorganisms12101940 (PMC11509545; doi:10.3390/microorganisms12101940)

Table S1. Nitrogen level and glucose addition on the greenhouse gas emissions rate

**Variables**	**Nitrogen level（N）**		**glucose（G）**		**N × G**	
	$F_{2,18}$	P	$F_{1,18}$	P	$F_{2,18}$	P
**Day 1**						
CH_4	1.99	0.180	1.14	0.306	0.23	0.796
CO_2	3.27	0.073	2.94	0.112	0.25	0.486
N_2O	**23.33**	**<0.001**	1.47	0.249	1.64	0.235
**Day 2**						
CH_4	**15.25**	**0.001**	**75.57**	**<0.001**	3.64	0.058
CO_2	1.08	0.370	**32.63**	**<0.001**	0.26	0.772
N_2O	**20.09**	**<0.001**	0.004	0.953	0.93	0.423
**Day 3**						
CH_4	**6.29**	**0.014**	**50.09**	**<0.001**	3.76	0.054
CO_2	1.00	0.395	**129.19**	**<0.001**	1.00	0.395
N_2O	**9.39**	**0.004**	0.04	0.839	0.16	0.854
**Day 5**						
CH_4	2.82	0.100	**77.74**	**<0.001**	1.85	0.200
CO_2	0.89	0.437	**186.82**	**<0.001**	0.12	0.892
N_2O	**7.19**	**0.009**	0.22	0.651	0.002	0.998
**Day 6**						
CH_4	3.17	0.078	**104.63**	**<0.001**	2.12	0.163
CO_2	2.56	0.118	**201.58**	**<0.001**	0.39	0.684
N_2O	**6.83**	**0.010**	0.43	0.528	0.04	0.962
**Day 7**						
CH_4	1.44	0.275	**144.99**	**<0.001**	0.95	0.414
CO_2	0.85	0.453	**330.91**	**<0.001**	0.39	0.686
N_2O	**5.81**	**0.017**	1.27	0.282	0.35	0.709

Notes: The given are F, degree of freedom and P of two-way ANOVAs. Values with $P < 0.05$ are in bold.

Table S2. Nitrogen level and glucose addition on the relative abundance of bacterial communities (Phylum) in the sediments

**Variables**	**Nitrogen level（N）**		**glucose（G）**		**N × G**	
	$F_{2,18}$	P	$F_{1,18}$	P	$F_{2,18}$	P
Firmicutes	2.06	0.170	**285.48**	**<0.001**	1.51	0.260
Proteobacteria	1.53	0.255	26.99	**<0.001**	2.30	0.143
Chloroflexi	1.03	0.387	**177.74**	**<0.001**	1.61	0.241
Actinobacteriota	3.84	0.051	**184.40**	**<0.001**	**5.90**	**0.016**
Bacteroidota	1.71	0.223	**23.57**	**<0.001**	0.85	0.451
Desulfobacterota	3.84	0.052	0.01	0.909	12.33	0.001
Acidobacteriota	0.40	0.677	**98.01**	**<0.001**	0.40	0.678
Myxococcota	0.16	0.857	**66.53**	**<0.001**	0.83	0.462

Notes: The given are *F*, degree of freedom and *P* of two-way ANOVAs. Values with $P < 0.05$ are in bold.

Figure S1. Effects of nitrogen and glucose addition on the nitrate nitrogen (a) and dissolved organic carbon contents (b) in the solution. Different capital letters indicate significant differences among nitrogen levels.

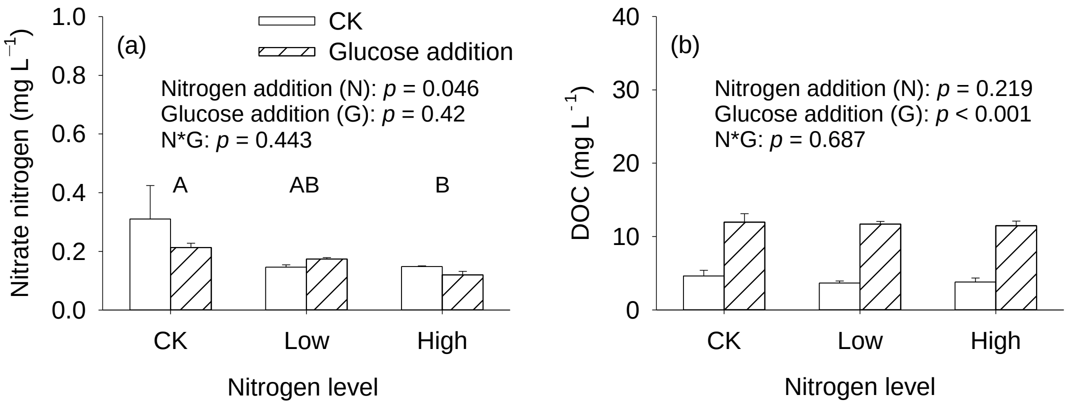

Figure S2. FAPROTAX of microbial function prediction. The abscissa is the sample name, and the ordinate is the function name.

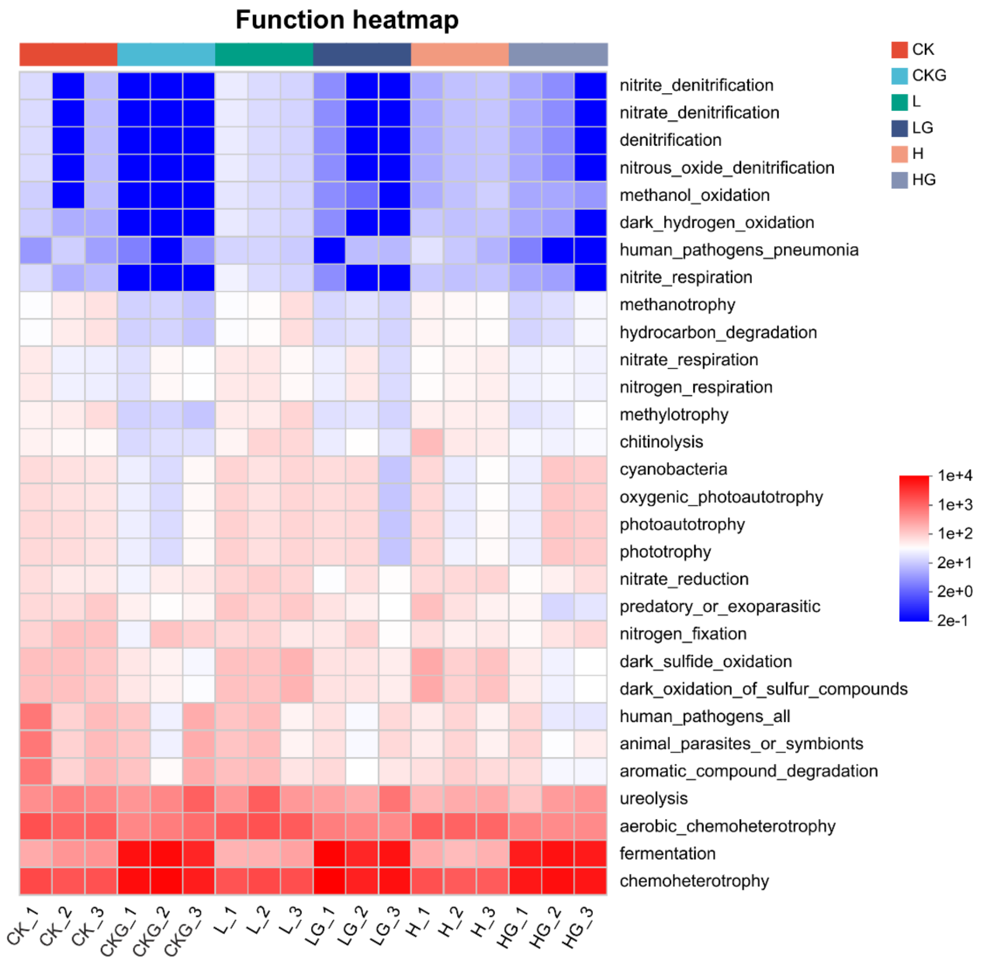

Supplement: Supplementary file 1 [file microorganisms-12-01940-s001.zip › microorganisms-3192913-supplementary.pdf]
